# Prevalence and predictors of frailty among community-dwelling older adults with hypertension in China

**DOI:** 10.1590/1806-9282.20250997

**Published:** 2025-12-05

**Authors:** Bai Chen, Yan Jiang, Siti Khuzaimah Ahmad Sharoni, Rosuzeita Fauzi, Siti Nor Ismalina Isa

**Affiliations:** 1 Universiti Teknologi MARA, Centre for Nursing Studies Selangor, Puncak Alam Campus, Faculty of Health Sciences – Puncak Alam, Malaysia.; 2 Capital Medical University, Xuanwu Hospital, Office of Clinical Research Administration – Beijing, China.; 3 Universiti Teknologi MARA, Faculty of Health Sciences, Department of Basic Sciences, Puncak Alam Campus – Puncak Alam, Malaysia.

**Keywords:** Frailty, Aged, Frail elderly, Hypertension, China

## Abstract

**OBJECTIVE:**

The aim of the study was to determine the prevalence, socio-demographic, and health-related predictors of frailty among community-dwelling elderly Chinese with hypertension.

**METHODS:**

It's a cross-sectional survey design among 338 respondents; the Chinese version of the Tilburg Frailty Assessment Scale was used to screen elderly hypertensive adults for frailty. The socio-demographic data were collected using a self-developed questionnaire. Data collected were analyzed using binary logistic regressions at a of 95%CI.

**RESULTS:**

The result of the study shows that the prevalence of frailty among community-dwelling older adults with hypertension in Henan province was 32.5%. The result of the socio-demographic predictors was being a female (p=0.001, OR 3.669 [CI 1.967–6.843]) and living in a rural area (p=0.032, OR 3.487 [CI 1.116–10.898]). While the leading health predictors were found to be a comorbidity of three or more conditions (p=0.001, OR 57.233 [CI 21.883–149.689]); history of hypertension spanning longer than 11 years (p=0.001, OR 10.541 [CI 5.404–20.561]).

**CONCLUSION:**

More attention should be focused on elderly hypertensive women to reduce the impact.

## INTRODUCTION

The aging population is expected to continue for the next three decades^1,2^. China is reported to have the highest number of older populations globally^3^. According to reports, the growing number of older population results from improved health outcomes and reduced causes of mortality^4^. As people age, evidence shows that they age with various physical and health challenges^2^. Frailty is one of the challenges that the older adult population experiences, which often leads to loss of self-sufficiency^5^. Frailty is a syndrome that is often accompanied by loss of weight, body weakness, and reduced level of activity^6^. The prevalence of frailty is reported to vary significantly based on factors, among which is the prevalence of chronic diseases^7^.

Hypertension increases the risk of debilitating diseases by causing various complications. The presence of multiple subclinical conditions and comorbidities accelerates the decline of physiological reserves across multiple body systems, disrupting age-related homeostasis and ultimately leading to frailty^8^. Furthermore, evidence shows that elderly hypertensive patients who are frail tend to have a poorer prognosis.

There is a high prevalence of poorly managed hypertension in Henan Province^9^. This is supported by the fact that evidence reported that a good number of the Chinese population are not just elderly but also hypertensive^10^. Evidence shows that hypertension not only impacts the cardiovascular system but also impairs the ability of elderly individuals to perform activities of daily living (ADL). With a decline in ADL, muscle strength declines and predisposes the individual to physical frailty^8,10^.

Despite these worrisome statistics, to the researcher's knowledge, to date, there is no validly acknowledged prevalence of frailty among the elderly population of Henan province. Additionally, to the researcher's knowledge, the socio-demographic and health predictors of frailty among community-dwelling hypertensives are not known^11^. Hence, this study was designed to determine the prevalence of frailty, social, and health-related predictors of frailty among community-dwelling elderly Chinese with hypertension in Henan Province.

## METHODS

### Study design, participants, and setting

The study is a cross-sectional survey conducted among community-dwelling, elderly, and hypertensive adults attending Zhou Kou Specialized Disease Hospital in Henan province.

### Eligibility criteria

Inclusion criteria include: elderly hypertensives who are above 60 years old, communicate effectively in Mandarin, with blood pressure greater than 140 and/or 90 mmHg. On the other hand, alcohol addicts, unconsciousness, and/or psychotic-like disorders are excluded.

### Outcomes, instruments, diagnostic criteria, or data collection

The outcome for this study was the prevalence of frailty, socio-demographic predictors, and health-related predictors of frailty among community-dwelling elderly Chinese with hypertension in Henan Province. The data collection instrument comprises 16 questions on basic information covering health and demographic characteristics, which were developed by the researchers with support from previous studies. Additionally, the Chinese version of the Tilburg Frailty Assessment Scale, which contains 15 questions, was used to screen elderly hypertensive adults with frailty. The scale has the physical, psychological, and social components. The score ranges from 0 to 15, with the scores of 0–8, 0–4, and 0–3 for the physical, psychological, and social components, respectively. The internal consistency of the Chinese version of the Tilburg Frailty Assessment Scale was Cronbach's α=0.71.

For blood pressure determination, a validated Omron automatic medical sphygmomanometer was used^12^. To take the blood pressure, all the clients are allowed to sit and relax for at least 5 min before the measurement. Thereafter, the cuff is placed on the patient's right arm about 2 cm above the elbow and fastened. The client's arms were supported on the table so that the arms are at the heart level and the start button is on. Clients are advised to remain calm during the reading until the cuff deflates and the reading is taken.

For measuring the weight, height, and body mass index (BMI), the DHM-800S ultrasonic height and weight measuring instrument was used. The instrument uses an ultrasonic probe to measure patients’ height and a precise sensor for measuring weight. The measurements of height, weight, and BMI are then automatically displayed on the screen.

### Bias

Healthcare workers who monitored the blood pressure were blinded to minimize bias.

### Sample, sample size calculation and sampling methods

For sample size calculation, the formula n=Z² * P(1-P)/d², as recommended^13^, was used with the prevalence of 67.6% from a study^14^, resulting in 338 samples.

### Ethical considerations

Ethical approval for this study was obtained from the Universiti Teknologi MARA Research Ethics Committee with approval number R.E.C./04/2023 (PG/MR/127) and Zhou Kou Specialized Disease Hospital. All participants read and completed informed consent forms. Participants were informed that they could withdraw from the study at any time if they felt uncomfortable while answering the questions.

### Data analysis

Data were analyzed using frequency, percentages, and mean. Additionally, binary logistic regression was used to determine the predictors of frailty at a 95%CI. Findings are regarded as statistically significant if the p<0.05. BMI was classified according to the Asian Pacific classification of BMI, which was used to classify obesity^15^.

## RESULTS

Table 1 shows the social and health-related characteristics of respondents. The mean age of the respondents was 65.99±4.98, with a somewhat equal distribution in terms of gender. Additionally, the majority (59.6%) of the respondents were married, with 93.8% residing in countryside, 50.3% reportedly revealed they had never smoked, and 48.4% never consumed alcohol as of the time of data collection. Additionally, the majority (41.6%) of the respondents only occasionally exercise, 46.6% have two comorbidities, 43.8% have a history of hypertensive disorder up to 5 years, and 45.6% reported polypharmacy. Furthermore, data reveals that the majority (70.9%) of the respondents were within normal BMI, while 32.5% were found to be frail.

**Table 1:** Social and health-related characteristics of respondents (n=320).

Variable		Frequency (n)	Percentage (%)
Age (years), M±SD	65.99±4.98		
Gender	Male	162	50.6
	Female	158	49.4
MHI Status (RMB), M±SD	6,768.56±2755.64 (942.671 USD)*		
Marital status	Married	190	59.6
	Widow	56	17.6
	Divorced	25	7.8
	Cohabitation	49	15.3
Place of residence	City	300	93.8
	Countryside	20	6.2
Smoking status	Yes, every day	8	2.5
	Yes, not daily	91	28.4
	Have quit	60	18.8
	Never	161	50.3
Alcohol	Yes, occasionally	97	30.3
	Have quit	68	21.3
	Never	155	48.4
Exercise	Never	48	15.0
	Occasionally	133	41.6
	Less than three times/week	101	31.6
	More than three times/week	38	11.9
Comorbidity (aside from hypertension)	1	110	34.4
	2	149	46.6
	3 and above	61	19.1
History of blood pressure (years)	1–5	140	43.8
	6–10	103	32.2
	≥11	77	24.0
Low-salt diet	Yes	34	10.6
	No	286	89.4
Sleep status	Not good	42	13.1
	Normal	257	80.3
	Very good	21	6.6
History of surgery	Yes	92	28.8
	No	228	71.2
Polypharmacy	Yes	146	45.6
	No	174	54.4
BMI, M±SD	Underweight	6	1.9
	Normal weight	227	70.9
	Overweight	84	26.3
	Obesity	3	0.9
Level of BP	Mild	154	48.1
	Moderate	115	36.0
	Severe	51	15.9
Frail status	Non-frail	216	67.5
	Frail	104	32.5

* 1USD=0.13927 RMD as at 10/11/2024; BMI: body mass index; BP: blood pressure; SD: standard deviation.

Results on the socio-demographic predictor of frailty, as presented in Table 2, show that age (p=0.001, OR 1.33 [CI 1.232–1.442]) being female [p=0.001, OR 3.669 (CI 1.967–6.843)], widow [p=0.0032, OR 0.497 (CI 0.262–0.942)], cohabiting [p=0.004, OR 0.047 (CI 0.006–0.376)] and living in the countryside [p=0.032, OR 3.487 (CI 1.116–10.898)] were found to be statistically significant in predicting frailty among older hypertensives.

**Table 2:** Sociodemographic predictors for frailty (n=320).

Predictors		B	OR	95%CI		Sig
				Lower	Upper	
Age (years)		0.287	1.333	1.232	1.442	0.001
Age category	60–65	0	1			
	66–70	4.124	0.016	0.004	0.074	0.001
	71–80	-2.127	0.119	0.026	0.546	0.006
	>80	-1.961	0.141	0.028	0.704	0.017
Gender	Male	0	1			
	Female	1.300	3.669	1.967	6.843	0.001
Marital status	Married	0	1			
	Widow	-0.700	0.497	0.262	0.942	0.032
	Divorced	-0.313	0.731	0.337	1.589	0.43
	Cohabitation	-3.055	0.047	0.006	0.376	0.004
Place of residence	City	0	1			
	Countryside	1.249	3.487	1.116	10.898	0.032

B: binary logistic regression; OR: odds ratio; CI: confidence interval.

Results show that diastolic BP, abstinence from alcohol and smoking, occasional exercise, nature of sleep, presence of comorbidity, polypharmacy, history of surgery, and low-salt diet were health-related predictors of frailty among community-dwelling older adults with hypertension. Details of the findings are shown in Table 3.

**Table 3:** Health-related predictors of frailty (n=320).

Predictors		B	OR	95%CI		Sig.
				Lower	Upper	
Systolic BP		-0.020	0.98	0.941	1.021	0.330
Diastolic BP		0.085	1.088	1.01	1.172	0.025
Level of BP	Mild	0	1			
	Moderate	1.6	4.952	2.768	8.862	0.001
	Severe	2.148	8.571	4.183	17.564	0.001
Smoking	Yes, every day	0	1			
	Yes, not daily	1.634	5.124	0.001	43,847.536	0.724
	Have quit	-1.508	0.221	0.032	1.535	0.127
	Never	-2.251	0.105	0.016	0.673	0.017
Exercise	Never	0	1			
	Occasionally	0.782	2.186	1.075	4.443	0.031
	Less than three times	-0.940	0.391	0.169	0.905	0.028
	More than three times/week	0.233	1.263	0.506	3.152	0.617
Alcohol	Yes, occasionally	0	1			
	Have quit	0.673	1.961	0.949	4.052	0.069
	Never	1.154	3.17	1.734	5.794	0.001
Sleep status	Not good	0	1			
	Normal	-2.632	0.072	0.031	0.170	0.001
	Good	-4.605	0.010	0.001	0.087	0.001
Comorbidity	1	0	1			
	2	1.548	4.703	2.183	10.129	0.001
	3 and above	4.047	57.233	21.883	149.689	0.001
History of hypertension	1–5	0	1			
	6–10	1.273	3.570	1.903	6.698	0.001
	>11	2.355	10.541	5.404	20.561	0.001
Polypharmacy	Yes	0	1			
	No	-2.519	0.081	0.045	0.145	0.001
History of surgery	Yes	0	1			
	No	-1.551	0.212	0.126	0.356	0.001
Low salt diet	Yes	0	1			
	No	2.183	8.87	2.083	37.770	0.003

B: binary logistic regression; OR: odds ratio; CI: confidence interval; BP: blood pressure.

## DISCUSSION

The findings of this study revealed a high prevalence of frailty among hypertensive older adults attending Zhou Kou Specialized Disease Hospital, Henan Province of China. This prevalence was found to be three-fold higher than the prevalence of frailty recorded in a systematic review and meta-analysis^16^. Additionally, the findings of this study are higher when compared to a national cross-sectional study conducted, where the researchers found a prevalence rate of frailty to be lower^17^. Therefore, the high prevalence found in this study when compared to the prevalence in similar studies^16,17^ gives a good indication as to the role comorbidity plays in the development of frailty; in this case, hypertension.

With regard to socio-demographic predictors of frailty among community-dwelling elderly Chinese with hypertension, study findings show that being a female, staying in rural areas, and widowhood are social predictors of frailty. These findings are consistent with a national cross-sectional survey involving 208,386 respondents across China^17^. The national cross-sectional study also reported that being female and living in rural areas was a major determinant of frailty^17^. Hormones such as estrogen during menopause could be associated with frailty among women^18^. With regard to marital status, results indicate that widowhood and cohabitation were associated with frailty. This finding is in line with previous studies, which indicate that elderly who are not living with their husbands are more likely to develop frailty and disability^17,18^. These might be because of limited support that these elderly have when they are either widowed or cohabitating.

Findings regarding the health-related predictors of frailty among community-dwelling elderly Chinese with hypertension reveal that the presence of three or more commodities and a history of hypertension are the predominant health-related predictors of frailty. The findings regarding commodities are partly consistent with evidence that reported a higher risk factor for frailty among respondents with comorbidities in their separate study^11,19^. According to reports, comorbid diseases are a significant determinant of health outcomes, especially among the older population^20^. Additionally, evidence also opined that a positive relationship exists between frailty and multiple comorbidities among the elderly population^21^. This is a consequence of reduced functional reserve and the continuous buildup of disease processes. In addition to these health-related factors, lifestyle behaviors such as dietary habits also critically affect the health status of elderly hypertensive patients. Most respondents in this study fail to follow low-salt dietary guidelines crucial for managing hypertension, posing significant health risks due to excessive salt intake. This highlights a critical need for targeted education and support to improve dietary compliance and health outcomes among elderly hypertensive patients.

## CONCLUSION

The findings show that there is a high prevalence of frailty among community-dwelling older adults with hypertension and emphasize the need to adequately screen and manage hypertension among the elderly population to slow and possibly prevent the onset of frailty.

## Data Availability

The datasets generated and/or analyzed during the current study are available from the corresponding author upon reasonable request.
